# Supplementary material for: Partition Function Zeros of the Frustrated J1–J2 Ising Model on the Honeycomb Lattice
Source: Entropy (Basel). 2024 Oct 29;26(11):919. doi: 10.3390/e26110919 (PMC11593144; doi:10.3390/e26110919)
Supplement: Supplementary file 1 [file entropy-26-00919-s001.zip › supplementaryMaterial.pdf]

# Supplementary Materials: Partition Function Zeros of the Frustrated $J_1$-$J_2$ Ising Model on the Honeycomb Lattice

**Denis Gessert, Martin Weigel and Wolfhard Janke**

## Contents


1. Video description . . . 1

2. All FSS fitting results . . . 1
   - 2.1. Temperature exponent $y_t$ . . . 2
     - 2.1.1. From the imaginary part of the Fisher zeros . . . 2
     - 2.1.2. From ordinary FSS . . . 4
   - 2.2. Determining the inverse critical temperature $\beta_c$ from the real part of the Fisher zeros . . . 5
   - 2.3. Field exponent $y_h$ . . . 7
     - 2.3.1. From the imaginary part of the Lee-Yang zeros . . . 8
     - 2.3.2. From ordinary FSS . . . 10


### 1. Video description

The supplementary video file shows the Fisher zeros of the $L = 4$ system for $\mathcal{R}$ continuously varying from 0 to $-1$ as determined by numerically solving for the roots of the polynomial, analogous to Figure 1 of the main text. Note that our Mathematica script fails to find zeros very close to the real axis. Hence, throughout the animation some zeros appear and disappear. This includes the leading zero which disappears for $\mathcal{R}$ close to $-1/4$. By numerically evaluating Equation (6) of the main text, we confirmed that the zero does not actually disappear, but rather approaches the origin as $\mathcal{R}$ goes to $-1/4$.

### 2. All FSS fitting results

In this section we provide tables with the details of all performed FSS fits for varying fit intervals from $L_{\mathrm{min}}$ to $L_{\mathrm{max}}$. In all tables $\chi^2_\nu$ denotes the $\chi^2$ value per degree of freedom computed from the error-weighted fits.

### 2.1. *Temperature exponent $y_t$*

#### 2.1.1. From the imaginary part of the Fisher zeros

**Table S1.** Fitting parameters of FSS fits using the ansatz $\Im(\beta_0(L)) = aL^{-y_t}$ for $\mathcal{R} = -0.1$ and different fitting ranges.

| **L**$_{\max}$ / **L**$_{\min}$ | 24 | 32 | 48 | 64 | 88 |
|---|---|---|---|---|---|
| 8 | $a = 1.249(17)$<br>$y_t = 0.9668(61)$<br>$\chi^2_\nu = 0.26$ | $a = 1.249(14)$<br>$y_t = 0.9667(48)$<br>$\chi^2_\nu = 0.13$ | $a = 1.285(10)$<br>$y_t = 0.9796(31)$<br>$\chi^2_\nu = 4.25$ | $a = 1.2908(99)$<br>$y_t = 0.9817(29)$<br>$\chi^2_\nu = 3.97$ | $a = 1.2942(98)$<br>$y_t = 0.9829(29)$<br>$\chi^2_\nu = 4.21$ |
| 16 | – | $a = 1.269(60)$<br>$y_t = 0.972(15)$<br>$\chi^2_\nu = 0.13$ | $a = 1.380(37)$<br>$y_t = 1.0001(79)$<br>$\chi^2_\nu = 2.34$ | $a = 1.391(33)$<br>$y_t = 1.0026(70)$<br>$\chi^2_\nu = 1.72$ | $a = 1.402(33)$<br>$y_t = 1.0052(68)$<br>$\chi^2_\nu = 2.00$ |
| 24 | – | – | $a = 1.467(71)$<br>$y_t = 1.017(13)$<br>$\chi^2_\nu = 2.35$ | $a = 1.472(61)$<br>$y_t = 1.018(11)$<br>$\chi^2_\nu = 1.18$ | $a = 1.495(60)$<br>$y_t = 1.022(11)$<br>$\chi^2_\nu = 1.38$ |
| 32 | – | – | – | $a = 1.62(13)$<br>$y_t = 1.042(21)$<br>$\chi^2_\nu = 0.36$ | $a = 1.66(12)$<br>$y_t = 1.049(19)$<br>$\chi^2_\nu = 0.57$ |
| 48 | – | – | – | – | $a = 1.65(25)$<br>$y_t = 1.048(38)$<br>$\chi^2_\nu = 1.14$ |

**Table S2.** As Table S1 but for $\mathcal{R} = -0.2$.

| **L**$_{\max}$ / **L**$_{\min}$ | 24 | 32 | 48 | 64 | 88 |
|---|---|---|---|---|---|
| 8 | $a = 2.856(25)$<br>$y_t = 0.9215(35)$<br>$\chi^2_\nu = 1.72$ | $a = 2.888(23)$<br>$y_t = 0.9263(31)$<br>$\chi^2_\nu = 5.25$ | $a = 2.960(20)$<br>$y_t = 0.9369(26)$<br>$\chi^2_\nu = 15.93$ | $a = 2.982(18)$<br>$y_t = 0.9400(23)$<br>$\chi^2_\nu = 13.61$ | $a = 3.021(17)$<br>$y_t = 0.9453(21)$<br>$\chi^2_\nu = 17.73$ |
| 16 | – | $a = 3.097(77)$<br>$y_t = 0.9500(86)$<br>$\chi^2_\nu = 1.58$ | $a = 3.244(52)$<br>$y_t = 0.9663(54)$<br>$\chi^2_\nu = 3.78$ | $a = 3.221(42)$<br>$y_t = 0.9639(42)$<br>$\chi^2_\nu = 2.71$ | $a = 3.281(37)$<br>$y_t = 0.9702(37)$<br>$\chi^2_\nu = 4.17$ |
| 24 | – | – | $a = 3.59(14)$<br>$y_t = 0.994(12)$<br>$\chi^2_\nu = 0.13$ | $a = 3.40(10)$<br>$y_t = 0.9780(83)$<br>$\chi^2_\nu = 2.13$ | $a = 3.502(86)$<br>$y_t = 0.9872(68)$<br>$\chi^2_\nu = 2.58$ |
| 32 | – | – | – | $a = 3.26(19)$<br>$y_t = 0.968(15)$<br>$\chi^2_\nu = 3.58$ | $a = 3.52(15)$<br>$y_t = 0.988(11)$<br>$\chi^2_\nu = 3.87$ |
| 48 | – | – | – | – | $a = 3.54(29)$<br>$y_t = 0.990(20)$<br>$\chi^2_\nu = 7.72$ |

**Table S3.** As Table S1 but for $\mathcal{R} = -0.21$.

| $\mathbf{L_{max}}$ / $\mathbf{L_{min}}$ | 24 | 32 | 48 | 64 | 88 |
|---|---|---|---|---|---|
| 8 | $a = 3.609(24)$ <br> $y_t = 0.9238(28)$ <br> $\chi^2_\nu = 5.52$ | $a = 3.628(21)$ <br> $y_t = 0.9262(24)$ <br> $\chi^2_\nu = 4.11$ | $a = 3.663(18)$ <br> $y_t = 0.9304(19)$ <br> $\chi^2_\nu = 5.58$ | $a = 3.693(16)$ <br> $y_t = 0.9340(17)$ <br> $\chi^2_\nu = 9.27$ | $a = 3.708(16)$ <br> $y_t = 0.9357(16)$ <br> $\chi^2_\nu = 9.75$ |
| 16 | – | $a = 3.859(86)$ <br> $y_t = 0.9461(73)$ <br> $\chi^2_\nu = 0.03$ | $a = 3.870(55)$ <br> $y_t = 0.9470(45)$ <br> $\chi^2_\nu = 0.03$ | $a = 3.937(48)$ <br> $y_t = 0.9527(38)$ <br> $\chi^2_\nu = 1.92$ | $a = 3.965(45)$ <br> $y_t = 0.9550(35)$ <br> $\chi^2_\nu = 2.05$ |
| 24 | – | – | $a = 3.87(10)$ <br> $y_t = 0.9471(77)$ <br> $\chi^2_\nu = 0.05$ | $a = 4.008(85)$ <br> $y_t = 0.9575(61)$ <br> $\chi^2_\nu = 2.36$ | $a = 4.053(77)$ <br> $y_t = 0.9609(53)$ <br> $\chi^2_\nu = 2.03$ |
| 32 | – | – | – | $a = 4.21(18)$ <br> $y_t = 0.970(11)$ <br> $\chi^2_\nu = 3.00$ | $a = 4.26(15)$ <br> $y_t = 0.9737(93)$ <br> $\chi^2_\nu = 1.64$ |
| 48 | – | – | – | – | $a = 4.70(33)$ <br> $y_t = 0.998(17)$ <br> $\chi^2_\nu = 0.59$ |

**Table S4.** As Table S1 but for $\mathcal{R} = -0.22$.

| $\mathbf{L_{max}}$ / $\mathbf{L_{min}}$ | 24 | 32 | 48 | 64 | 88 |
|---|---|---|---|---|---|
| 8 | $a = 5.088(34)$ <br> $y_t = 0.9328(30)$ <br> $\chi^2_\nu = 3.39$ | $a = 5.118(28)$ <br> $y_t = 0.9356(23)$ <br> $\chi^2_\nu = 2.77$ | $a = 5.128(26)$ <br> $y_t = 0.9364(21)$ <br> $\chi^2_\nu = 2.15$ | $a = 5.179(23)$ <br> $y_t = 0.9410(18)$ <br> $\chi^2_\nu = 5.59$ | $a = 5.239(20)$ <br> $y_t = 0.9461(15)$ <br> $\chi^2_\nu = 9.45$ |
| 16 | – | $a = 5.40(13)$ <br> $y_t = 0.9525(76)$ <br> $\chi^2_\nu = 0.04$ | $a = 5.38(11)$ <br> $y_t = 0.9513(63)$ <br> $\chi^2_\nu = 0.06$ | $a = 5.503(80)$ <br> $y_t = 0.9588(45)$ <br> $\chi^2_\nu = 1.00$ | $a = 5.597(62)$ <br> $y_t = 0.9644(32)$ <br> $\chi^2_\nu = 1.56$ |
| 24 | – | – | $a = 5.31(22)$ <br> $y_t = 0.948(13)$ <br> $\chi^2_\nu = 0.01$ | $a = 5.60(14)$ <br> $y_t = 0.9634(71)$ <br> $\chi^2_\nu = 1.16$ | $a = 5.714(97)$ <br> $y_t = 0.9697(46)$ <br> $\chi^2_\nu = 1.24$ |
| 32 | – | – | – | $a = 5.77(24)$ <br> $y_t = 0.971(11)$ <br> $\chi^2_\nu = 1.37$ | $a = 5.88(16)$ <br> $y_t = 0.9766(68)$ <br> $\chi^2_\nu = 0.87$ |
| 48 | – | – | – | – | $a = 6.35(45)$ <br> $y_t = 0.995(16)$ <br> $\chi^2_\nu = 0.32$ |

#### 2.1.2. From ordinary FSS

**Table S5.** Fitting parameters of FSS fits using the ansatz $\mathrm{d}\ln|m|_{\max}(L) = aL^{y_t}$ for $\mathcal{R} = -0.1$ and different fitting ranges.

| $\mathbf{L}_{\max}$ <br> $\mathbf{L}_{\min}$ | 24 | 32 | 48 | 64 | 88 |
|---|---|---|---|---|---|
| 8 | $a = 0.6599(42)$ <br> $y_t = 0.9957(24)$ <br> $\chi^2_\nu = 0.99$ | $a = 0.6619(33)$ <br> $y_t = 0.9943(17)$ <br> $\chi^2_\nu = 0.82$ | $a = 0.6623(30)$ <br> $y_t = 0.9941(15)$ <br> $\chi^2_\nu = 0.57$ | $a = 0.6627(28)$ <br> $y_t = 0.9939(13)$ <br> $\chi^2_\nu = 0.45$ | $a = 0.6624(24)$ <br> $y_t = 0.9941(11)$ <br> $\chi^2_\nu = 0.37$ |
| 16 | – | $a = 0.679(22)$ <br> $y_t = 0.9867(96)$ <br> $\chi^2_\nu = 0.98$ | $a = 0.670(12)$ <br> $y_t = 0.9908(51)$ <br> $\chi^2_\nu = 0.62$ | $a = 0.6681(82)$ <br> $y_t = 0.9916(35)$ <br> $\chi^2_\nu = 0.43$ | $a = 0.6644(57)$ <br> $y_t = 0.9932(23)$ <br> $\chi^2_\nu = 0.42$ |
| 24 | – | – | $a = 0.670(12)$ <br> $y_t = 0.9908(51)$ <br> $\chi^2_\nu = 0.25$ | $a = 0.6681(82)$ <br> $y_t = 0.9916(35)$ <br> $\chi^2_\nu = 0.15$ | $a = 0.6644(57)$ <br> $y_t = 0.9933(23)$ <br> $\chi^2_\nu = 0.23$ |
| 32 | – | – | – | $a = 0.663(12)$ <br> $y_t = 0.9935(50)$ <br> $\chi^2_\nu = 0.01$ | $a = 0.6602(78)$ <br> $y_t = 0.9948(31)$ <br> $\chi^2_\nu = 0.05$ |
| 48 | – | – | – | – | $a = 0.657(18)$ <br> $y_t = 0.9959(65)$ <br> $\chi^2_\nu = 0.07$ |

**Table S6.** As Table S5 but for $\mathcal{R} = -0.2$.

| $\mathbf{L}_{\max}$ <br> $\mathbf{L}_{\min}$ | 24 | 32 | 48 | 64 | 88 |
|---|---|---|---|---|---|
| 8 | $a = 0.3174(21)$ <br> $y_t = 0.9211(27)$ <br> $\chi^2_\nu = 9.65$ | $a = 0.3123(15)$ <br> $y_t = 0.9284(18)$ <br> $\chi^2_\nu = 11.55$ | $a = 0.3078(13)$ <br> $y_t = 0.9344(15)$ <br> $\chi^2_\nu = 21.90$ | $a = 0.3051(12)$ <br> $y_t = 0.9380(14)$ <br> $\chi^2_\nu = 25.60$ | $a = 0.29727(97)$ <br> $y_t = 0.9484(10)$ <br> $\chi^2_\nu = 47.13$ |
| 16 | – | $a = 0.2918(44)$ <br> $y_t = 0.9493(47)$ <br> $\chi^2_\nu = 0.00$ | $a = 0.2841(32)$ <br> $y_t = 0.9581(34)$ <br> $\chi^2_\nu = 3.77$ | $a = 0.2803(27)$ <br> $y_t = 0.9625(29)$ <br> $\chi^2_\nu = 4.35$ | $a = 0.2730(18)$ <br> $y_t = 0.9707(19)$ <br> $\chi^2_\nu = 6.60$ |
| 24 | – | – | $a = 0.2729(61)$ <br> $y_t = 0.9694(64)$ <br> $\chi^2_\nu = 3.15$ | $a = 0.2689(46)$ <br> $y_t = 0.9737(48)$ <br> $\chi^2_\nu = 2.10$ | $a = 0.2643(26)$ <br> $y_t = 0.9788(26)$ <br> $\chi^2_\nu = 1.92$ |
| 32 | – | – | – | $a = 0.2597(62)$ <br> $y_t = 0.9829(65)$ <br> $\chi^2_\nu = 0.00$ | $a = 0.2593(33)$ <br> $y_t = 0.9833(32)$ <br> $\chi^2_\nu = 0.00$ |
| 48 | – | – | – | – | $a = 0.2588(73)$ <br> $y_t = 0.9838(67)$ <br> $\chi^2_\nu = 0.00$ |

**Table S7.** As Table S5 but for $\mathcal{R} = -0.21$.

| $\mathbf{L}_{\text{max}}$ / $\mathbf{L}_{\text{min}}$ | 24 | 32 | 48 | 64 | 88 |
|---|---|---|---|---|---|
| 8 | $a = 0.2733(23)$ $y_t = 0.8953(31)$ $\chi^2_\nu = 15.57$ | $a = 0.2590(15)$ $y_t = 0.9170(19)$ $\chi^2_\nu = 46.27$ | $a = 0.2505(11)$ $y_t = 0.9294(14)$ $\chi^2_\nu = 63.30$ | $a = 0.2484(11)$ $y_t = 0.9323(13)$ $\chi^2_\nu = 55.68$ | $a = 0.24342(96)$ $y_t = 0.9392(12)$ $\chi^2_\nu = 69.06$ |
| 16 | – | $a = 0.2401(24)$ $y_t = 0.9405(31)$ $\chi^2_\nu = 4.61$ | $a = 0.2342(16)$ $y_t = 0.9488(20)$ $\chi^2_\nu = 8.41$ | $a = 0.2328(15)$ $y_t = 0.9508(19)$ $\chi^2_\nu = 7.25$ | $a = 0.2284(12)$ $y_t = 0.9567(16)$ $\chi^2_\nu = 13.99$ |
| 24 | – | – | $a = 0.2214(34)$ $y_t = 0.9642(43)$ $\chi^2_\nu = 0.00$ | $a = 0.2209(28)$ $y_t = 0.9648(35)$ $\chi^2_\nu = 0.03$ | $a = 0.2161(21)$ $y_t = 0.9711(26)$ $\chi^2_\nu = 2.21$ |
| 32 | – | – | – | $a = 0.2206(36)$ $y_t = 0.9652(44)$ $\chi^2_\nu = 0.05$ | $a = 0.2146(24)$ $y_t = 0.9727(30)$ $\chi^2_\nu = 2.72$ |
| 48 | – | – | – | – | $a = 0.2064(46)$ $y_t = 0.9822(55)$ $\chi^2_\nu = 1.33$ |

**Table S8.** As Table S5 but for $\mathcal{R} = -0.22$.

| $\mathbf{L}_{\text{max}}$ / $\mathbf{L}_{\text{min}}$ | 24 | 32 | 48 | 64 | 88 |
|---|---|---|---|---|---|
| 8 | $a = 0.2122(14)$ $y_t = 0.8781(25)$ $\chi^2_\nu = 50.78$ | $a = 0.2060(11)$ $y_t = 0.8907(19)$ $\chi^2_\nu = 54.35$ | $a = 0.20043(87)$ $y_t = 0.9019(15)$ $\chi^2_\nu = 70.52$ | $a = 0.19573(76)$ $y_t = 0.9112(13)$ $\chi^2_\nu = 86.54$ | $a = 0.19291(71)$ $y_t = 0.9167(12)$ $\chi^2_\nu = 95.88$ |
| 16 | – | $a = 0.1819(24)$ $y_t = 0.9302(42)$ $\chi^2_\nu = 0.00$ | $a = 0.1785(16)$ $y_t = 0.9365(28)$ $\chi^2_\nu = 2.01$ | $a = 0.1750(13)$ $y_t = 0.9431(22)$ $\chi^2_\nu = 5.70$ | $a = 0.1721(11)$ $y_t = 0.9484(19)$ $\chi^2_\nu = 11.45$ |
| 24 | – | – | $a = 0.1740(33)$ $y_t = 0.9437(54)$ $\chi^2_\nu = 1.53$ | $a = 0.1689(22)$ $y_t = 0.9525(36)$ $\chi^2_\nu = 3.22$ | $a = 0.1645(18)$ $y_t = 0.9600(30)$ $\chi^2_\nu = 7.05$ |
| 32 | – | – | – | $a = 0.1632(33)$ $y_t = 0.9612(53)$ $\chi^2_\nu = 1.47$ | $a = 0.1580(26)$ $y_t = 0.9702(42)$ $\chi^2_\nu = 4.60$ |
| 48 | – | – | – | – | $a = 0.1452(50)$ $y_t = 0.9905(84)$ $\chi^2_\nu = 1.47$ |

### *2.2. Determining the inverse critical temperature $\beta_c$ from the real part of the Fisher zeros*

With the limited number of data points, a three-parameter fit for the real part of the Fisher zero is rather unstable. Therefore, we fix $y_t = 1$ in the fit ansatz $\Re(\beta_0(L)) = \beta_c - aL^{-y_t} - bL^{-2y_t}$. Results are shown in Tables S9 to S12.

**Table S9.** Fitting parameters of FSS fits using the ansatz $\Re(\beta_0(L)) = \beta_c - aL^{-y_t} - bL^{-2y_t}$ ($y_t = 1$ fixed) for $\mathcal{R} = -0.1$ and different fitting ranges.

| **L**$_{\text{max}}$ / **L**$_{\text{min}}$ | 32 | 48 | 64 | 88 |
|---|---|---|---|---|
| 8 | $a = 0.279(41)$<br>$b = 1.13(25)$<br>$\beta_c = 1.0226(13)/J_1$<br>$\chi^2_\nu = 0.29$ | $a = 0.310(21)$<br>$b = 0.95(15)$<br>$\beta_c = 1.02371(52)/J_1$<br>$\chi^2_\nu = 0.55$ | $a = 0.301(19)$<br>$b = 1.00(14)$<br>$\beta_c = 1.02344(43)/J_1$<br>$\chi^2_\nu = 0.65$ | $a = 0.304(16)$<br>$b = 0.99(12)$<br>$\beta_c = 1.02350(32)/J_1$<br>$\chi^2_\nu = 0.50$ |
| 16 | – | $a = 0.351(75)$<br>$b = 0.44(92)$<br>$\beta_c = 1.0244(13)/J_1$<br>$\chi^2_\nu = 0.79$ | $a = 0.300(59)$<br>$b = 1.02(75)$<br>$\beta_c = 1.02342(94)/J_1$<br>$\chi^2_\nu = 0.98$ | $a = 0.309(41)$<br>$b = 0.92(55)$<br>$\beta_c = 1.02357(58)/J_1$<br>$\chi^2_\nu = 0.66$ |
| 24 | – | – | $a = 0.27(15)$<br>$b = 1.6(2.6)$<br>$\beta_c = 1.0230(20)/J_1$<br>$\chi^2_\nu = 1.89$ | $a = 0.307(79)$<br>$b = 1.0(1.5)$<br>$\beta_c = 1.02355(91)/J_1$<br>$\chi^2_\nu = 1.00$ |
| 32 | – | – | – | $a = 0.22(14)$<br>$b = 3.4(3.3)$<br>$\beta_c = 1.0228(13)/J_1$<br>$\chi^2_\nu = 1.29$ |

**Table S10.** As Table S9 but for $\mathcal{R} = -0.2$.

| **L**$_{\text{max}}$ / **L**$_{\text{min}}$ | 32 | 48 | 64 | 88 |
|---|---|---|---|---|
| 8 | $a = 0.813(62)$<br>$b = 6.64(38)$<br>$\beta_c = 2.6563(20)/J_1$<br>$\chi^2_\nu = 0.01$ | $a = 0.803(34)$<br>$b = 6.70(23)$<br>$\beta_c = 2.65588(85)/J_1$<br>$\chi^2_\nu = 0.02$ | $a = 0.841(30)$<br>$b = 6.47(21)$<br>$\beta_c = 2.65706(72)/J_1$<br>$\chi^2_\nu = 2.22$ | $a = 0.853(25)$<br>$b = 6.40(18)$<br>$\beta_c = 2.65740(54)/J_1$<br>$\chi^2_\nu = 1.79$ |
| 16 | – | $a = 0.79(11)$<br>$b = 6.9(1.4)$<br>$\beta_c = 2.6556(20)/J_1$<br>$\chi^2_\nu = 0.03$ | $a = 0.940(92)$<br>$b = 5.2(1.2)$<br>$\beta_c = 2.6586(15)/J_1$<br>$\chi^2_\nu = 2.68$ | $a = 0.932(64)$<br>$b = 5.26(87)$<br>$\beta_c = 2.65843(94)/J_1$<br>$\chi^2_\nu = 1.79$ |
| 24 | – | – | $a = 1.29(25)$<br>$b = -1.0(4.2)$<br>$\beta_c = 2.6630(33)/J_1$<br>$\chi^2_\nu = 3.02$ | $a = 1.04(13)$<br>$b = 3.1(2.4)$<br>$\beta_c = 2.6596(15)/J_1$<br>$\chi^2_\nu = 2.21$ |
| 32 | – | – | – | $a = 1.12(22)$<br>$b = 1.1(5.3)$<br>$\beta_c = 2.6603(22)/J_1$<br>$\chi^2_\nu = 4.23$ |

**Table S11.** As Table S9 but for $\mathcal{R} = -0.21$.

| $\mathbf{L}_{\max}$ / $\mathbf{L}_{\min}$ | 32 | 48 | 64 | 88 |
|---|---|---|---|---|
| 8 | $a = 1.010(65)$<br>$b = 9.91(39)$<br>$\beta_c = 3.2539(23)/J_1$<br>$\chi^2_\nu = 4.80$ | $a = 1.040(31)$<br>$b = 9.74(22)$<br>$\beta_c = 3.25504(91)/J_1$<br>$\chi^2_\nu = 2.54$ | $a = 1.056(25)$<br>$b = 9.65(19)$<br>$\beta_c = 3.25560(64)/J_1$<br>$\chi^2_\nu = 1.94$ | $a = 1.106(20)$<br>$b = 9.34(17)$<br>$\beta_c = 3.25721(42)/J_1$<br>$\chi^2_\nu = 4.22$ |
| 16 | – | $a = 1.22(13)$<br>$b = 7.6(1.6)$<br>$\beta_c = 3.2581(24)/J_1$<br>$\chi^2_\nu = 3.13$ | $a = 1.208(97)$<br>$b = 7.7(1.2)$<br>$\beta_c = 3.2578(15)/J_1$<br>$\chi^2_\nu = 1.58$ | $a = 1.314(64)$<br>$b = 6.48(86)$<br>$\beta_c = 3.25965(83)/J_1$<br>$\chi^2_\nu = 1.76$ |
| 24 | – | – | $a = 0.94(27)$<br>$b = 12.6(4.7)$<br>$\beta_c = 3.2545(34)/J_1$<br>$\chi^2_\nu = 2.00$ | $a = 1.36(13)$<br>$b = 5.6(2.6)$<br>$\beta_c = 3.2601(14)/J_1$<br>$\chi^2_\nu = 2.57$ |
| 32 | – | – | – | $a = 1.81(24)$<br>$b = -7.3(6.3)$<br>$\beta_c = 3.2637(22)/J_1$<br>$\chi^2_\nu = 0.18$ |

**Table S12.** As Table S9 but for $\mathcal{R} = -0.22$.

| $\mathbf{L}_{\max}$ / $\mathbf{L}_{\min}$ | 32 | 48 | 64 | 88 |
|---|---|---|---|---|
| 8 | $a = 1.565(76)$<br>$b = 13.85(50)$<br>$\beta_c = 4.2722(22)/J_1$<br>$\chi^2_\nu = 1.14$ | $a = 1.481(44)$<br>$b = 14.36(33)$<br>$\beta_c = 4.26966(98)/J_1$<br>$\chi^2_\nu = 1.47$ | $a = 1.511(37)$<br>$b = 14.17(29)$<br>$\beta_c = 4.27045(77)/J_1$<br>$\chi^2_\nu = 1.55$ | $a = 1.500(31)$<br>$b = 14.25(25)$<br>$\beta_c = 4.27017(59)/J_1$<br>$\chi^2_\nu = 1.24$ |
| 16 | – | $a = 1.40(12)$<br>$b = 15.5(1.5)$<br>$\beta_c = 4.2683(20)/J_1$<br>$\chi^2_\nu = 2.39$ | $a = 1.518(90)$<br>$b = 14.1(1.2)$<br>$\beta_c = 4.2705(14)/J_1$<br>$\chi^2_\nu = 2.32$ | $a = 1.486(65)$<br>$b = 14.46(93)$<br>$\beta_c = 4.27000(94)/J_1$<br>$\chi^2_\nu = 1.63$ |
| 24 | – | – | $a = 1.49(21)$<br>$b = 14.6(3.8)$<br>$\beta_c = 4.2702(27)/J_1$<br>$\chi^2_\nu = 4.62$ | $a = 1.44(13)$<br>$b = 15.5(2.5)$<br>$\beta_c = 4.2695(15)/J_1$<br>$\chi^2_\nu = 2.36$ |
| 32 | – | – | – | $a = 1.65(20)$<br>$b = 10.2(4.6)$<br>$\beta_c = 4.2715(21)/J_1$<br>$\chi^2_\nu = 2.84$ |

### 2.3. *Field exponent $y_h$*

We use either the imaginary part of the Lee-Yang zeros or the magnetic susceptibility to obtain $y_h$, in both cases considering the system directly at the infinite-volume inverse critical temperature $\beta_c$. As described in the main text, the error bars for $y_h$ obtained after fixing $\beta_c$ at the values of Tables S9-S12 using FSS fits do not account for the uncertainty in $\beta_c$. Therefore, we use jackknifing over the whole process to correctly include both the statistical error in $\beta_c$ as well as that in the Lee-Yang zeros and the susceptibility, respectively.

#### 2.3.1. From the imaginary part of the Lee-Yang zeros

**Table S13.** Fitting parameters of FSS fits at $\beta_c$ using the ansatz $\Im(h_0(L)) = aL^{-y_h}$ for $\mathcal{R} = -0.1$ and different fitting ranges, using the jackknife procedure described in the main text.

| $\mathbf{L}_{\max}$ / $\mathbf{L}_{\min}$ | 24 | 32 | 48 | 64 | 88 |
|---|---|---|---|---|---|
| 8 | $a = 0.7329(11)$<br>$y_h = 1.87629(97)$<br>$\chi^2_\nu = 0.22(56)$ | $a = 0.73178(82)$<br>$y_h = 1.87564(69)$<br>$\chi^2_\nu = 1.6(1.7)$ | $a = 0.7317(17)$<br>$y_h = 1.8756(13)$<br>$\chi^2_\nu = 1.4(1.9)$ | $a = 0.7313(28)$<br>$y_h = 1.8754(20)$<br>$\chi^2_\nu = 1.5(2.6)$ | $a = 0.7313(38)$<br>$y_h = 1.8754(25)$<br>$\chi^2_\nu = 1.5(3.1)$ |
| 16 | – | $a = 0.7297(26)$<br>$y_h = 1.8747(13)$<br>$\chi^2_\nu = 2.3(1.8)$ | $a = 0.7305(41)$<br>$y_h = 1.8751(23)$<br>$\chi^2_\nu = 1.6(3.0)$ | $a = 0.7302(55)$<br>$y_h = 1.8750(30)$<br>$\chi^2_\nu = 1.2(3.0)$ | $a = 0.7306(67)$<br>$y_h = 1.8751(35)$<br>$\chi^2_\nu = 1.1(3.4)$ |
| 24 | – | – | $a = 0.7290(80)$<br>$y_h = 1.8745(38)$<br>$\chi^2_\nu = 2.6(6.3)$ | $a = 0.7294(91)$<br>$y_h = 1.8747(42)$<br>$\chi^2_\nu = 1.4(3.5)$ | $a = 0.730(10)$<br>$y_h = 1.8750(46)$<br>$\chi^2_\nu = 1.1(2.6)$ |
| 32 | – | – | – | $a = 0.733(11)$<br>$y_h = 1.8758(49)$<br>$\chi^2_\nu = 0.7(1.6)$ | $a = 0.733(11)$<br>$y_h = 1.8757(50)$<br>$\chi^2_\nu = 0.42(88)$ |
| 48 | – | – | – | – | $a = 0.731(11)$<br>$y_h = 1.8752(49)$<br>$\chi^2_\nu = 0.4(1.3)$ |

**Table S14.** As Table S13 but for $\mathcal{R} = -0.2$.

| $\mathbf{L}_{\max}$ / $\mathbf{L}_{\min}$ | 24 | 32 | 48 | 64 | 88 |
|---|---|---|---|---|---|
| 8 | $a = 0.26075(58)$<br>$y_h = 1.8780(13)$<br>$\chi^2_\nu = 0.8(4.3)$ | $a = 0.26028(72)$<br>$y_h = 1.8772(16)$<br>$\chi^2_\nu = 2.1(4.1)$ | $a = 0.26021(83)$<br>$y_h = 1.8771(18)$<br>$\chi^2_\nu = 1.5(3.1)$ | $a = 0.26013(95)$<br>$y_h = 1.8769(20)$<br>$\chi^2_\nu = 1.5(3.6)$ | $a = 0.2600(15)$<br>$y_h = 1.8767(30)$<br>$\chi^2_\nu = 1.9(4.7)$ |
| 16 | – | $a = 0.2584(16)$<br>$y_h = 1.8750(26)$<br>$\chi^2_\nu = 0.6(2.0)$ | $a = 0.2590(17)$<br>$y_h = 1.8757(28)$<br>$\chi^2_\nu = 0.62(87)$ | $a = 0.2590(21)$<br>$y_h = 1.8757(32)$<br>$\chi^2_\nu = 0.47(65)$ | $a = 0.2592(30)$<br>$y_h = 1.8759(44)$<br>$\chi^2_\nu = 0.51(73)$ |
| 24 | – | – | $a = 0.2592(24)$<br>$y_h = 1.8759(35)$<br>$\chi^2_\nu = 1.0(1.8)$ | $a = 0.2590(26)$<br>$y_h = 1.8757(38)$<br>$\chi^2_\nu = 0.64(88)$ | $a = 0.2593(36)$<br>$y_h = 1.8760(48)$<br>$\chi^2_\nu = 0.54(77)$ |
| 32 | – | – | – | $a = 0.2598(30)$<br>$y_h = 1.8765(41)$<br>$\chi^2_\nu = 0.5(1.4)$ | $a = 0.2596(40)$<br>$y_h = 1.8763(52)$<br>$\chi^2_\nu = 0.35(88)$ |
| 48 | – | – | – | – | $a = 0.2591(55)$<br>$y_h = 1.8758(66)$<br>$\chi^2_\nu = 0.3(1.4)$ |

**Table S15.** As Table S13 but for $\mathcal{R} = -0.21$.

| $\mathbf{L_{max}}$ / $\mathbf{L_{min}}$ | 24 | 32 | 48 | 64 | 88 |
|---|---|---|---|---|---|
| 8 | $a = 0.21298(68)$<br>$y_h = 1.8826(16)$<br>$\chi^2_\nu = 0.17(52)$ | $a = 0.21264(67)$<br>$y_h = 1.8819(17)$<br>$\chi^2_\nu = 1.8(2.1)$ | $a = 0.21235(79)$<br>$y_h = 1.8813(20)$<br>$\chi^2_\nu = 1.7(2.0)$ | $a = 0.21240(90)$<br>$y_h = 1.8814(22)$<br>$\chi^2_\nu = 1.4(1.4)$ | $a = 0.2125(14)$<br>$y_h = 1.8817(30)$<br>$\chi^2_\nu = 1.5(2.0)$ |
| 16 | – | $a = 0.2114(14)$<br>$y_h = 1.8799(27)$<br>$\chi^2_\nu = 1.6(2.2)$ | $a = 0.2116(13)$<br>$y_h = 1.8803(26)$<br>$\chi^2_\nu = 0.9(1.2)$ | $a = 0.2119(14)$<br>$y_h = 1.8807(29)$<br>$\chi^2_\nu = 1.0(1.4)$ | $a = 0.2124(20)$<br>$y_h = 1.8814(37)$<br>$\chi^2_\nu = 1.5(2.3)$ |
| 24 | – | – | $a = 0.2112(17)$<br>$y_h = 1.8798(30)$<br>$\chi^2_\nu = 1.4(2.1)$ | $a = 0.2119(20)$<br>$y_h = 1.8807(35)$<br>$\chi^2_\nu = 1.5(2.0)$ | $a = 0.2127(27)$<br>$y_h = 1.8818(44)$<br>$\chi^2_\nu = 1.6(2.0)$ |
| 32 | – | – | – | $a = 0.2143(25)$<br>$y_h = 1.8836(38)$<br>$\chi^2_\nu = 0.21(69)$ | $a = 0.2139(30)$<br>$y_h = 1.8831(48)$<br>$\chi^2_\nu = 0.26(77)$ |
| 48 | – | – | – | – | $a = 0.2139(39)$<br>$y_h = 1.8832(57)$<br>$\chi^2_\nu = 0.2(1.1)$ |

**Table S16.** As Table S13 but for $\mathcal{R} = -0.22$.

| $\mathbf{L_{max}}$ / $\mathbf{L_{min}}$ | 24 | 32 | 48 | 64 | 88 |
|---|---|---|---|---|---|
| 8 | $a = 0.16181(28)$<br>$y_h = 1.88294(89)$<br>$\chi^2_\nu = 1.1(1.8)$ | $a = 0.16140(38)$<br>$y_h = 1.8818(11)$<br>$\chi^2_\nu = 3.7(2.9)$ | $a = 0.16102(42)$<br>$y_h = 1.8808(12)$<br>$\chi^2_\nu = 6.2(3.5)$ | $a = 0.16083(36)$<br>$y_h = 1.8804(10)$<br>$\chi^2_\nu = 5.3(2.5)$ | $a = 0.16081(36)$<br>$y_h = 1.8803(10)$<br>$\chi^2_\nu = 4.3(1.9)$ |
| 16 | – | $a = 0.1592(11)$<br>$y_h = 1.8777(22)$<br>$\chi^2_\nu = 1.1(2.2)$ | $a = 0.15869(85)$<br>$y_h = 1.8766(16)$<br>$\chi^2_\nu = 1.0(1.4)$ | $a = 0.15944(62)$<br>$y_h = 1.8781(14)$<br>$\chi^2_\nu = 1.6(1.7)$ | $a = 0.15948(58)$<br>$y_h = 1.8781(13)$<br>$\chi^2_\nu = 1.2(1.3)$ |
| 24 | – | – | $a = 0.15792(83)$<br>$y_h = 1.8752(17)$<br>$\chi^2_\nu = 0.14(64)$ | $a = 0.15935(84)$<br>$y_h = 1.8779(18)$<br>$\chi^2_\nu = 2.2(2.5)$ | $a = 0.15941(76)$<br>$y_h = 1.8780(17)$<br>$\chi^2_\nu = 1.6(1.7)$ |
| 32 | – | – | – | $a = 0.1602(12)$<br>$y_h = 1.8792(23)$<br>$\chi^2_\nu = 2.5(3.2)$ | $a = 0.1602(11)$<br>$y_h = 1.8793(21)$<br>$\chi^2_\nu = 1.3(1.7)$ |
| 48 | – | – | – | – | $a = 0.1625(22)$<br>$y_h = 1.8827(36)$<br>$\chi^2_\nu = 0.7(1.9)$ |

#### 2.3.2. From ordinary FSS

**Table S17.** Fitting parameters of FSS fits at $\beta_c$ using the ansatz $\chi_L(\beta_c) = aL^{2y_h - D}$ for $\mathcal{R} = -0.1$ and different fitting ranges, using the jackknife procedure described in the main text. $D = 2$ is the spatial dimension.

| $\mathbf{L}_{\max}$ / $\mathbf{L}_{\min}$ | 24 | 32 | 48 | 64 | 88 |
|---|---|---|---|---|---|
| 8 | $a = 2.3235(60)$<br>$y_h = 1.87813(86)$<br>$\chi^2_\nu = 0.20(47)$ | $a = 2.3324(45)$<br>$y_h = 1.87730(57)$<br>$\chi^2_\nu = 3.9(3.4)$ | $a = 2.3364(88)$<br>$y_h = 1.8769(11)$<br>$\chi^2_\nu = 3.6(3.3)$ | $a = 2.342(16)$<br>$y_h = 1.8764(17)$<br>$\chi^2_\nu = 4.5(5.5)$ | $a = 2.344(21)$<br>$y_h = 1.8763(21)$<br>$\chi^2_\nu = 4.2(5.2)$ |
| 16 | – | $a = 2.359(14)$<br>$y_h = 1.8755(11)$<br>$\chi^2_\nu = 2.7(2.0)$ | $a = 2.356(23)$<br>$y_h = 1.8757(20)$<br>$\chi^2_\nu = 1.7(3.0)$ | $a = 2.360(31)$<br>$y_h = 1.8754(26)$<br>$\chi^2_\nu = 1.4(2.5)$ | $a = 2.358(38)$<br>$y_h = 1.8755(31)$<br>$\chi^2_\nu = 1.3(2.9)$ |
| 24 | – | – | $a = 2.367(46)$<br>$y_h = 1.8751(34)$<br>$\chi^2_\nu = 2.7(6.5)$ | $a = 2.367(51)$<br>$y_h = 1.8750(37)$<br>$\chi^2_\nu = 1.4(3.4)$ | $a = 2.363(57)$<br>$y_h = 1.8753(40)$<br>$\chi^2_\nu = 1.1(2.7)$ |
| 32 | – | – | – | $a = 2.351(62)$<br>$y_h = 1.8759(42)$<br>$\chi^2_\nu = 1.0(1.9)$ | $a = 2.351(63)$<br>$y_h = 1.8759(43)$<br>$\chi^2_\nu = 0.6(1.0)$ |
| 48 | – | – | – | – | $a = 2.361(64)$<br>$y_h = 1.8754(43)$<br>$\chi^2_\nu = 0.7(1.7)$ |

**Table S18.** As Table S17 but for $\mathcal{R} = -0.2$.

| $\mathbf{L}_{\max}$ / $\mathbf{L}_{\min}$ | 24 | 32 | 48 | 64 | 88 |
|---|---|---|---|---|---|
| 8 | $a = 6.944(31)$<br>$y_h = 1.8828(13)$<br>$\chi^2_\nu = 5(13)$ | $a = 6.990(37)$<br>$y_h = 1.8813(14)$<br>$\chi^2_\nu = 11(15)$ | $a = 7.013(43)$<br>$y_h = 1.8805(16)$<br>$\chi^2_\nu = 11(14)$ | $a = 7.027(48)$<br>$y_h = 1.8801(17)$<br>$\chi^2_\nu = 12(15)$ | $a = 7.067(85)$<br>$y_h = 1.8789(28)$<br>$\chi^2_\nu = 18(23)$ |
| 16 | – | $a = 7.202(79)$<br>$y_h = 1.8767(23)$<br>$\chi^2_\nu = 1.2(3.1)$ | $a = 7.200(86)$<br>$y_h = 1.8767(24)$<br>$\chi^2_\nu = 0.7(1.6)$ | $a = 7.21(10)$<br>$y_h = 1.8765(28)$<br>$\chi^2_\nu = 0.6(1.1)$ | $a = 7.22(15)$<br>$y_h = 1.8763(38)$<br>$\chi^2_\nu = 0.6(1.1)$ |
| 24 | – | – | $a = 7.23(12)$<br>$y_h = 1.8761(30)$<br>$\chi^2_\nu = 0.8(1.5)$ | $a = 7.24(13)$<br>$y_h = 1.8759(33)$<br>$\chi^2_\nu = 0.53(77)$ | $a = 7.23(18)$<br>$y_h = 1.8761(42)$<br>$\chi^2_\nu = 0.46(67)$ |
| 32 | – | – | – | $a = 7.21(15)$<br>$y_h = 1.8765(36)$<br>$\chi^2_\nu = 0.5(1.4)$ | $a = 7.22(20)$<br>$y_h = 1.8763(46)$<br>$\chi^2_\nu = 0.36(88)$ |
| 48 | – | – | – | – | $a = 7.24(27)$<br>$y_h = 1.8759(57)$<br>$\chi^2_\nu = 0.3(1.4)$ |

**Table S19.** As Table S17 but for $\mathcal{R} = -0.21$.

| $\mathbf{L_{max}}$ / $\mathbf{L_{min}}$ | 24 | 32 | 48 | 64 | 88 |
|---|---|---|---|---|---|
| 8 | $a = 8.488(44)$<br>$y_h = 1.8873(13)$<br>$\chi^2_\nu = 5.2(3.3)$ | $a = 8.537(37)$<br>$y_h = 1.8860(11)$<br>$\chi^2_\nu = 10.1(4.6)$ | $a = 8.627(55)$<br>$y_h = 1.8839(16)$<br>$\chi^2_\nu = 16.2(7.7)$ | $a = 8.637(61)$<br>$y_h = 1.8837(18)$<br>$\chi^2_\nu = 12.8(6.4)$ | $a = 8.669(95)$<br>$y_h = 1.8830(25)$<br>$\chi^2_\nu = 12.6(8.2)$ |
| 16 | – | $a = 8.795(97)$<br>$y_h = 1.8812(23)$<br>$\chi^2_\nu = 2.6(2.8)$ | $a = 8.819(92)$<br>$y_h = 1.8807(22)$<br>$\chi^2_\nu = 1.5(1.5)$ | $a = 8.81(10)$<br>$y_h = 1.8810(24)$<br>$\chi^2_\nu = 1.3(1.3)$ | $a = 8.79(15)$<br>$y_h = 1.8813(33)$<br>$\chi^2_\nu = 1.3(1.8)$ |
| 24 | – | – | $a = 8.89(12)$<br>$y_h = 1.8797(26)$<br>$\chi^2_\nu = 1.4(2.1)$ | $a = 8.84(14)$<br>$y_h = 1.8804(30)$<br>$\chi^2_\nu = 1.5(2.0)$ | $a = 8.79(20)$<br>$y_h = 1.8812(39)$<br>$\chi^2_\nu = 1.5(2.1)$ |
| 32 | – | – | – | $a = 8.68(17)$<br>$y_h = 1.8828(33)$<br>$\chi^2_\nu = 0.23(83)$ | $a = 8.72(22)$<br>$y_h = 1.8823(43)$<br>$\chi^2_\nu = 0.31(86)$ |
| 48 | – | – | – | – | $a = 8.72(28)$<br>$y_h = 1.8823(50)$<br>$\chi^2_\nu = 0.3(1.2)$ |

**Table S20.** As Table S17 but for $\mathcal{R} = -0.22$.

| $\mathbf{L_{max}}$ / $\mathbf{L_{min}}$ | 24 | 32 | 48 | 64 | 88 |
|---|---|---|---|---|---|
| 8 | $a = 11.203(30)$<br>$y_h = 1.88778(65)$<br>$\chi^2_\nu = 5.2(3.7)$ | $a = 11.292(53)$<br>$y_h = 1.8860(11)$<br>$\chi^2_\nu = 13.4(5.5)$ | $a = 11.373(67)$<br>$y_h = 1.8845(12)$<br>$\chi^2_\nu = 21.6(8.4)$ | $a = 11.460(56)$<br>$y_h = 1.88300(95)$<br>$\chi^2_\nu = 24.8(6.1)$ | $a = 11.467(55)$<br>$y_h = 1.88288(95)$<br>$\chi^2_\nu = 20.6(5.0)$ |
| 16 | – | $a = 11.81(15)$<br>$y_h = 1.8791(19)$<br>$\chi^2_\nu = 2.6(3.4)$ | $a = 11.92(13)$<br>$y_h = 1.8777(16)$<br>$\chi^2_\nu = 2.2(2.4)$ | $a = 11.853(87)$<br>$y_h = 1.8786(13)$<br>$\chi^2_\nu = 2.0(1.9)$ | $a = 11.851(83)$<br>$y_h = 1.8786(12)$<br>$\chi^2_\nu = 1.5(1.4)$ |
| 24 | – | – | $a = 12.07(12)$<br>$y_h = 1.8758(16)$<br>$\chi^2_\nu = 0.15(75)$ | $a = 11.89(11)$<br>$y_h = 1.8781(15)$<br>$\chi^2_\nu = 2.1(2.7)$ | $a = 11.89(10)$<br>$y_h = 1.8782(15)$<br>$\chi^2_\nu = 1.5(1.9)$ |
| 32 | – | – | – | $a = 11.79(15)$<br>$y_h = 1.8792(19)$<br>$\chi^2_\nu = 2.2(3.4)$ | $a = 11.79(14)$<br>$y_h = 1.8792(18)$<br>$\chi^2_\nu = 1.2(1.7)$ |
| 48 | – | – | – | – | $a = 11.54(27)$<br>$y_h = 1.8817(30)$<br>$\chi^2_\nu = 0.8(1.9)$ |

**Disclaimer/Publisher's Note:** The statements, opinions and data contained in all publications are solely those of the individual author(s) and contributor(s) and not of MDPI and/or the editor(s). MDPI and/or the editor(s) disclaim responsibility for any injury to people or property resulting from any ideas, methods, instructions or products referred to in the content.
